# Supplementary material for: Knowledge, Attitudes, and Practices Regarding Antibiotic Sales in Pharmacies in Medellín, Colombia 2023
Source: Antibiotics (Basel). 2023 Sep 19;12(9):1456. doi: 10.3390/antibiotics12091456 (PMC10525149; doi:10.3390/antibiotics12091456)
Supplement: Supplementary file 1 [file antibiotics-12-01456-s001.zip › antibiotics-2621153-supplementary.pdf]

Table S1: Multiple comparisons, adjusted with the Bonferroni test

Pairwise Comparisons of Zone

Sample 1-Sample 2	Test Statistic	Std. Error	Std. Test Statistic	Sig.	Adj. Sig.[a]
Southeastern zone - Rural districts	5.331	38.468	.139	.890	1.000
Southeastern zone - Southwestern zone	-27.946	18.983	-1.472	.141	1.000
Southeastern zone - Northeastern zone	42.943	20.657	2.079	.038	.790
Southeastern zone - Western-central	54.981	18.592	2.957	.003	.065
Southeastern zone - Eastern-central	56.014	18.496	3.028	.002	.052
Southeastern zone Northwestern zone	73.231	19.845	3.690	<.001	.005
Rural districts - Southwestern zone	-22.616	36.916	-.613	.540	1.000
Rural districts - Northeastern zone	-37.612	37.804	-.995	.320	1.000
Rural districts - Western-central	-49.650	36.717	-1.352	.176	1.000
Rural districts - Eastern-central	-50.683	36.668	-1.382	.167	1.000
Rural districts - Northwestern zone	-67.900	37.367	-1.817	.069	1.000
Southwestern zone - Northeastern zone	14.996	17.599	.852	.394	1.000
Southwestern zone - Western-central	27.034	15.122	1.788	.074	1.000
Southwestern zone - Eastern-central	28.068	15.004	1.871	.061	1.000
Southwestern zone - Northwestern zone	45.284	16.638	2.722	.006	.136
Northeastern zone - Western-central	-12.038	17.177	-.701	.483	1.000
Northeastern zone - Eastern-central	-13.071	17.073	-.766	.444	1.000

Northeastern zone - Northwestern zone	-30.288	18.525	-1.635	.102	1.000
Western-central - Eastern-central	1.033	14.506	.071	.943	1.000
Western-central - Northwestern zone	18.250	16.191	1.127	.260	1.000
Eastern-central - Northwestern zone	17.217	16.080	1.071	.284	1.000
Each row tests the null hypothesis that the Sample 1 and Sample 2 distributions are the same. Asymptotic significances (2-sided tests) are displayed. The significance level is .050.					
a. Significance values have been adjusted by the Bonferroni correction for multiple tests.					

Pairwise Comparisons of Studies

Sample 1-Sample 2	Test Statistic	Std. Error	Std. Test Statistic	Sig.	Adj. Sig.[a]
Pharmacy Assistant -Other	-27.507	15.726	-1.749	.080	.241
Pharmacy Assistant - Pharmacy Technician	42.855	10.258	4.178	<.001	.000
Other- Pharmacy Technician	15.348	16.037	.957	.339	1.000
Each row tests the null hypothesis that the Sample 1 and Sample 2 distributions are the same. Asymptotic significances (2-sided tests) are displayed. The significance level is .050.					
a. Significance values have been adjusted by the Bonferroni correction for multiple tests.					

Pairwise Comparisons of work experience

Sample 1-Sample 2	Test Statistic	Std. Error	Std. Test Statistic	Sig.	Adj. Sig.[a]
2 to 5 years - < 2 years	2.121	14.875	.143	.887	1.000
2 to 5 years - > 5 years	-34.808	11.911	-2.922	.003	.010
< 2 years - > 5 years	-32.687	12.483	-2.619	.009	.026
Each row tests the null hypothesis that the Sample 1 and Sample 2 distributions are the same. Asymptotic significances (2-sided tests) are displayed. The significance level is .050.					
a. Significance values have been adjusted by the Bonferroni correction for multiple tests.					

S2: Survey

Survey on the Knowledge, Attitudes, and Practices of Pharmacists Regarding the Use of Antibiotics and Antibiotic Resistance in Medellín.

The data requested in this survey are confidential, and your identity will not be requested at any time. The information obtained here will only be used for research purposes. The results will be published using statistical aggregates. We kindly ask you to respond honestly to the questions we will ask you, and please remember that your responses are anonymous.

Below, you will find some statements. Please rate your level of agreement/disagreement with them on a scale from one to four, like this: **1. Completely disagree**, **2. Disagree**, **3. Agree,** o **4. Completely agree**

	1	2	3	4
Everyone should take antibiotics every year.				
Antibiotics are used to treat stomach pain and diarrhea.				
Patients can stop taking antibiotics when their symptoms improve.				
Antibiotics are effective for treating the common cold, cough, and sore throat.				
Antibiotics are effective for treating Covid-19.				
Fever can be directly reduced with antibiotics.				
An antibiotic will always be effective in treating the same infection in the future.				
There are antibiotics that can be taken without medical prescription.				

Below, you will find some statements. Please rate your level of agreement/disagreement with them on a scale from one to four, like this: **1. Completely disagree**, **2. Disagree**, **3. Agree,** o **4. Completely agree**

	1	2	3	4
Pharmacists should be authorized to prescribe antibiotics for uncomplicated infections.				
The prohibition of selling antibiotics without a medical prescription will decrease pharmacy sales and profits.				
According to the medical prescriptions that arrive at your pharmacy, the prescription of antibiotics should be more closely monitored.				
The sale of antibiotics without a medical prescription should be more closely controlled.				
If I refuse to sell antibiotics to a patient who doesn't need them, they could easily obtain them from another pharmacy.				

Below, you will find some statements about certain practices. Please mark according to the frequency with which you perform them, like this: **1. Never, 2. rarely, 3. Almost always, o 4. Always**

	1	2	3	4
I have been encouraged by the pharmacy to sell antibiotics and receive a commission for each sale				
I recommend antibiotic treatment to patients with COVID-19 symptoms				

I have sold antibiotics without a medical prescription to patients with dental infections (e.g., abscess)				
I have sold antibiotics without a medical prescription to adult patients with undiagnosed infections.				
I have sold antibiotics without a medical prescription to patients with upper respiratory tract infections (e.g., otitis, pharyngitis).				
Antibiotics are sometimes dispensed without a medical prescription because the patient has difficulties in obtaining a consultation				
I have sold antibiotics without a medical prescription to patients with urinary tract infections				
I have sold fewer doses of antibiotics than indicated in the medical prescription at the patient's request.				
